# Decreased body mass index in the preclinical stage of autosomal dominant Alzheimer’s disease

**DOI:** 10.1038/s41598-017-01327-w

**Published:** 2017-04-27

**Authors:** Stephan Müller, Oliver Preische, Hamid R. Sohrabi, Susanne Gräber, Mathias Jucker, Janko Dietzsch, John M. Ringman, Ralph N. Martins, Eric McDade, Peter R. Schofield, Bernardino Ghetti, Martin Rossor, Neill R. Graff-Radford, Johannes Levin, Douglas Galasko, Kimberly A. Quaid, Stephen Salloway, Chengjie Xiong, Tammie Benzinger, Virginia Buckles, Colin L. Masters, Reisa Sperling, Randall J. Bateman, John C. Morris, Christoph Laske

**Affiliations:** 1 Department of Psychiatry and Psychotherapy, University of Tübingen, 72076 Tübingen, Germany; 2 German Center for Neurodegenerative Diseases (DZNE), 72076 Tübingen, Germany; 3 Centre of Excellence for Alzheimer’s Disease Research and Care, School of Medical Sciences, Edith Cowan University, Perth, WA 6027 Australia; 4 School of Psychiatry and Clinical Neurosciences, University of Western Australia, Nedlands, WA 6009 Australia; 5 Section for Dementia Research, Department of Cellular Neurology, Hertie Institute for Clinical Brain Research and Department of Psychiatry and Psychotherapy, University of Tübingen, 72076 Tübingen, Germany; 6 Department of Cellular Neurology, Hertie Institute for Clinical Brain Research, University of Tübingen, 72076 Tübingen, Germany; 7 Memory and Aging Center, Keck School of Medicine of USC, Los Angeles, CA USA; 8 University of Pittsburgh School of Medicine, Department of Neurology, 3471 5th Ave, Suite 811, Pittsburgh, PA 15213 USA; 9 Neuroscience Research Australia, Randwick, Sydney, NSW 2031 Australia; 10 School of Medical Sciences, University of New South Wales, Sydney, NSW 2052 Australia; 11 Department of Pathology and Laboratory Medicine, Indiana University, Indianapolis, IN 46202 USA; 12 Dementia Research Centre, Department of Neurodegeneration, UCL Institute of Neurology, Queen Square, London, WC1 3BG UK; 13 Department of Neuroscience, Mayo Clinic, Jacksonville, Florida and Department of Neurology, Mayo Clinic, Jacksonville, Florida USA; 14 German Center for Neurodegenerative Diseases (DZNE), München, Germany and Department of Neurology, Ludwig-Maximilians Universität Munich, Munich, Germany; 15 Shiley-Marcos Alzheimer’s Disease Research Center, Department of Neurosciences, University of California, San Diego, CA USA; 16 Indiana University Center for Bioethics, 410 West 10th Street, Indianapolis, IN 46202 USA; 17 Department of Neurology, The Warren Alpert Medical School of Brown University, Providence, RI USA; 18 Division of Biostatistics, The Knight Alzheimer’s Disease Research Center, Washington University School of Medicine, St Louis, MO USA; 19 Department of Neurology, Knight Alzheimer’s Disease Research Center, Washington University School of Medicine, St. Louis, MO 63108 USA; 20 Mental Health Research Institute, University of Melbourne, Level 5, Kenneth Myer Building, 30 Royal Parade, Parkville, Victoria, 3010 Australia; 21 Center for Alzheimer Research and Treatment, Department of Neurology, Brigham and Women’s Hospital, Massachusetts General Hospital, Harvard Medical School, Boston, MA USA

## Abstract

The relationship between body-mass index (BMI) and Alzheimer´s disease (AD) has been extensively investigated. However, BMI alterations in preclinical individuals with autosomal dominant AD (ADAD) have not yet been investigated. We analyzed cross-sectional data from 230 asymptomatic members of families with ADAD participating in the Dominantly Inherited Alzheimer Network (DIAN) study including 120 preclinical mutation carriers (MCs) and 110 asymptomatic non-carriers (NCs). Differences in BMI and their relation with cerebral amyloid load and episodic memory as a function of estimated years to symptom onset (EYO) were analyzed. Preclinical MCs showed significantly lower BMIs compared to NCs, starting 11.2 years before expected symptom onset. However, the BMI curves begun to diverge already at 17.8 years before expected symptom onset. Lower BMI in preclinical MCs was significantly associated with less years before estimated symptom onset, higher global Aβ brain burden, and with lower delayed total recall scores in the logical memory test. The study provides cross-sectional evidence that weight loss starts one to two decades before expected symptom onset of ADAD. Our findings point toward a link between the pathophysiology of ADAD and disturbance of weight control mechanisms. Longitudinal follow-up studies are warranted to investigate BMI changes over time.

## Introduction

The relationship between weight or body mass index (BMI) and late-onset Alzheimer’s disease (AD) has been extensively investigated. According to previous studies, the association between BMI and risk for future development of AD seems to depend on the time point of BMI assessment. While higher BMI in midlife has been shown to be a risk factor for future development of AD, higher BMI in late-life is associated with a reduced AD risk^1–3^. In contrast, low BMI and accelerated weight loss in later life have been associated with increased risk of AD^4–8^. Up to now, it is still unclear at what time point BMI decreases during the preclinical stage of AD.

To address these research questions, we examined cross-sectional data of asymptomatic autosomal-dominant AD (ADAD) family members participating in the Dominantly Inherited Alzheimer Network (DIAN)^9^. DIAN is a multi-center, international project studying a large cohort of individuals, aged 18 years and over, who are carriers of an AD-causing mutation (MCs) and their non-carrier (NCs) family members (http://www.dian-info.org). All participants have undertaken regular assessments at baseline and follow-up sessions including blood and cerebrospinal fluid (CSF) collection as well as clinical, neuropsychological and neuroimaging examinations. DIAN procedures, overall approach and structures have been previously described in more details^9, 10^.

MCs of ADAD develop the disease at a younger age due to mutations in amyloid precursor protein (APP) or presenilin genes (PSEN1, PSEN2) and usually in the absence of vascular and metabolic risk factors^11^. Thus, the study of MCs compared to NCs allows detecting preclinical BMI differences caused by AD-related neurodegenerative processes and not confounded by additional age-related factors. Furthermore, study of ADAD allows examining BMI across the whole spectrum of the disease beginning more than 20 years prior to the estimated symptom onset. In the present study we were interested (1) to examine the relationship between BMI and estimated years to expected symptom onset in preclinical ADAD and (2) whether BMI was associated with cognitive and imaging data in preclinical ADAD.

## Materials and Methods

### Participants

In this study, cross-sectional baseline data of 230 participants from the DIAN study were analyzed including 110 asymptomatic NCs (first-degree relatives of MCs) and 120 preclinical MCs consisting of 88 PSEN1 carriers, 14 PSEN2 carriers, and 18 APP carriers.

Further demographic information is provided in Table 1. Experimental protocols described in the present study have been approved by the Ethik-Kommission an der Medizinischen Fakultät der Eberhard-Karls-Universität und am Universitätsklinikum Tübingen. All other aspects of the study have been approved by the institutional review boards (IRB) for each of the participating sites in the DIAN. All methods were performed in accordance with the relevant guidelines and regulations. All participants provided written, informed consent.

**Table 1:** Baseline demographics, body mass index, clinical, cognitive, biochemical and imaging parameters in preclinical (CDR = 0) mutation carriers (MCs) and non-carriers (NCs).

	NCs (N = 110)	MCs (N = 120)	P-value
Age	36.7 (7.9)	35.3 (8.0)	0.238^a^
Gender (M/F) no.	49/72	64/88	0.789^b^
Education (years)	13.2 (0.4)	13.4 (0.6)	0.735^a^
GDS	1.3 (1.8)	1.6 (1.9)	0.248^c^
EYO	−11.8 (7.1)	−11.5 (6.7)	0.759^a^
BMI, kg/m^2^	30.1 (5.9)	26.5 (5.2)	**0.0001** ^a^
BMI < 18.5 (%)	1.1	2.1	0.984^b^
BMI ≥ 18.5 to <25.0 (%)	24.7	34.0	0.165^b^
BMI ≥ 25.0 to <30.0 (%)	30.3	38.1	0.263^b^
BMI ≥ 30 (%)	43.8	25.8	**0.009** ^b^
Stroke (%)	1.1	0	0.443^d^
Diabetes (%)	2.2	0	0.195^d^
Hypertension (%)	16.9	5.4	**0.010** ^d^
Hypercholesterolemia (%)	11.2	15.2	0.533^d^
MMSE score	29.1 (1.5)	29.1 (1.4)	0.695^a^
LogMem I	15.01 (4.2)	13.8 (4.8)	0.037^a^
LogMem II	13.9 (4.4)	12.03 (5.3)	**0.003** ^a^
Global PIB-uptake	1.03 (0.1)	1.7 (0.8)	**<0.0001** ^a^

Note: Displayed are means and standard deviations (SD); MCs = mutation carriers; NCs = non mutation carriers; ^a^ANCOVA comparisons at Bonferroni adjusted p < 0.006 level of significance; ^b^Pearson chi-square test; ^c^Mann-Whitney U test; ^d^Fisher’s exact test; CDR = Clinical Dementia Rating scale; M = male; F = female; GDS = Geriatric Depression Scale; BMI = body mass index; no. = number; % = percentage; EYO = estimated years to symptom onset; MMSE = Mini-Mental State Examination; LogMem I = Logical Memory test, immediate recall; LogMem II = Logical Memory test, delayed recall; Global PIB-uptake = global cerebral Aβ burden as measured by ^11^C-Pittsburgh Compound-B PET.

### Clinical assessments

Participants underwent clinical assessment of cognitive status using the Clinical Dementia Rating (CDR) global score scale. Only participants with no relevant clinical symptoms of cognitive impairment were included (i.e. CDR = 0)^12^.

Body mass index (BMI, [kg/m^2^]) for each participant at baseline visit was calculated from the height and weight and categorized as underweight (<18.5 kg/m^2^), normal (≥18.5 to <25.0 kg/m^2^), pre-obese (≥25.0 to <30.0 kg/m^2^), or obese (≥30.0 kg/m^2^) according to the World Health Organization (WHO) criteria^13^.

Presence or absence of vascular comorbidities (i.e. stroke, diabetes, hypertension, and hypercholesterolemia) was assessed at baseline by clinical interview. Estimated years from expected symptom onset (EYO) were calculated as the age of the participant at baseline assessment minus the age of their parent at symptom onset as previously described^11^. For example, if the participant’s age was 37 years, and the parent’s age at onset was 45 years, then the estimated years from expected symptom onset would be −8. As all participants of the DIAN study are members of affected ADAD families, the construct of EYO can be applied to both MCs and NCs, resulting in age-matched cases and controls. The EYO concept allows the use of cross-sectional data to gain insight into the disease trajectory over time and has been validated in the DIAN study as providing a highly accurate estimate of AD biomarkers staging and symptom onset^11, 14^.

All demographic data (age, gender, education, EYO), clinical assessments (CDR, Geriatric Depression Scale [GDS]), cognitive measurements (Mini-Mental State Examination [MMSE], logical memory subtest of the Wechsler Memory Scale-III), and global cerebral Aβ burden as measured by ^11^C-Pittsburgh Compound-B (PiB) PET were performed as recently described^11^.

### Data analysis

Between group differences in age, education, global cognition (MMSE), logical memory scores (immediate and delayed total word recall), BMI, EYO and global PiB-uptake between MCs und NCs were assessed using one-way analyses of variance controlling for participant age, education, gender, and participant family (ANCOVA). Continuous variables were examined to assess normality. Levene’s test was used to assess homogeneity of variance. The level of statistical significance was set to P < 0.05, two-tailed. In order to avoid alpha error accumulation, Bonferroni-correction was conducted (i.e. comparisons for this analysis were performed at the P < 0.006 level of significance). The Pearson chi-square test was used to detect group differences in gender and BMI categories (i.e. <18.5 kg/m^2^, ≥18.5 to <25.0 kg/m^2^, ≥25.0 to <30.0 kg/m^2^, ≥30.0 kg/m^2^) distribution and the nonparametric Mann-Whitney U test to detect group differences in GDS scores. Fisher’s exact test was used to detect incidence differences in vascular comorbidities (stroke, diabetes, hypertension, and hypercholesterolemia).

Relationships within NCs or MCs between BMI and AD-related brain biomarkers (i.e. global cerebral Aβ burden), EYO and psychometric parameters (i.e. MMSE and delayed total recall scores of the Wechsler Memory Scale-III) were examined using multiple linear regressions controlling for participant age, education and sex.

Relationships within NCs or MCs between BMI and AD-related brain biomarkers (i.e. global cerebral Aβ burden), EYO and psychometric parameters (i.e. MMSE and delayed total recall scores of the Wechsler Memory Scale-III) were examined using multiple linear regressions controlling for participant age, education and sex and with family cluster as a random effect. The point at which the association between BMI and EYO differs significantly between MCs and NCs corresponds to the area where the estimated 95% confidence intervals (CI) for the regression lines do not overlap. The intersection points of the lower and upper confidence interval borders were calculated by a numerical approximation. In addition, we used this method to detect the point, at which the two curves begin to diverge. Statistical analyses were conducted with self-written functions in R^15^ and the use of its packages particular for the visualization ggplot2^16^.

It should be noted that use of scatter plots is not permitted with DIAN data as the data points can be very specific to an individual and may allow to identify study participants’ mutation status.

## Results

### Demographics and clinical parameters in preclinical (CDR = 0) MCs and NCs of the DIAN study

MCs and NCs were comparable regarding age (P = 0.238), gender (P = 0.789), and educational level (P = 0.735), showing no significant differences. Also, GDS scores were not significantly different between groups (P = 0.248). Although depressive symptoms were reported by both MC and NC individuals, it is not until a GDS score of greater or equal 6 indicates clinical relevant mild depression. There were significantly (P = 0.009) more obese NCs (i.e. BMI ≥ 30) and more NCs with hypertension compared to MCs (Table 1).

Mean BMI and number of preclinical (CDR = 0) MCs and NCs divided in 5-year time segments from −25 to 0 EYO are displayed in Table 2.

**Table 2:** Mean body mass index (BMI) and number of preclinical (CDR = 0) mutation carriers (MCs) and non-mutation carriers (NCs) in 5-year time segments from −25 to 0 years of estimated symptom onset (EYO).

BMI at EYO/no of MCs or NCs	NCs (N = 110)	MCs (N = 120)
−25.0 to −20.1/no	28.6 (25.1–32.2)/15	28.4 (25.6–31.1)/16
−20.0 to −15.1/no	29.4 (26.7–32.1)/24	26.4 (23.7–29.0)/22
−15.0 to −10.1/no	31.7 (28.8–35.1)/25	25.4 (23.2–27.6)/29
−10.0 to −5.1/no	31.9 (28.3–35.6)/24	26.5 (24.7–28.3)/28
−5.0 to 0.0/no	31.1 (26.4–33.7)/22	25.9 (23.7–28.1)/25

Note: Displayed are means and 95% confidence intervals (in parentheses) of body mass index (BMI) and the number of MCs and NCs in 5-year time segments starting from −25 to 0 years of EYO; CDR = Clinical Dementia Rating scale.

### Cognitive and imaging parameters of preclinical (CDR = 0) participants of the DIAN study

MCs with CDR = 0 showed significantly higher global cerebral Aβ burden (P < 0.0001) and reduced delayed total recall (P = 0.003) in the logical memory test as compared to NCs. However, memory scores were within normal ranges in both groups. Immediate total recall scores in the logical memory test and MMSE scores did not differ between preclinical (CDR = 0) MCs and NCs (Table 1).

### Association between BMI, cognition, and imaging parameters in preclinical (CDR = 0) MCs and NCs

**Figure 1:**
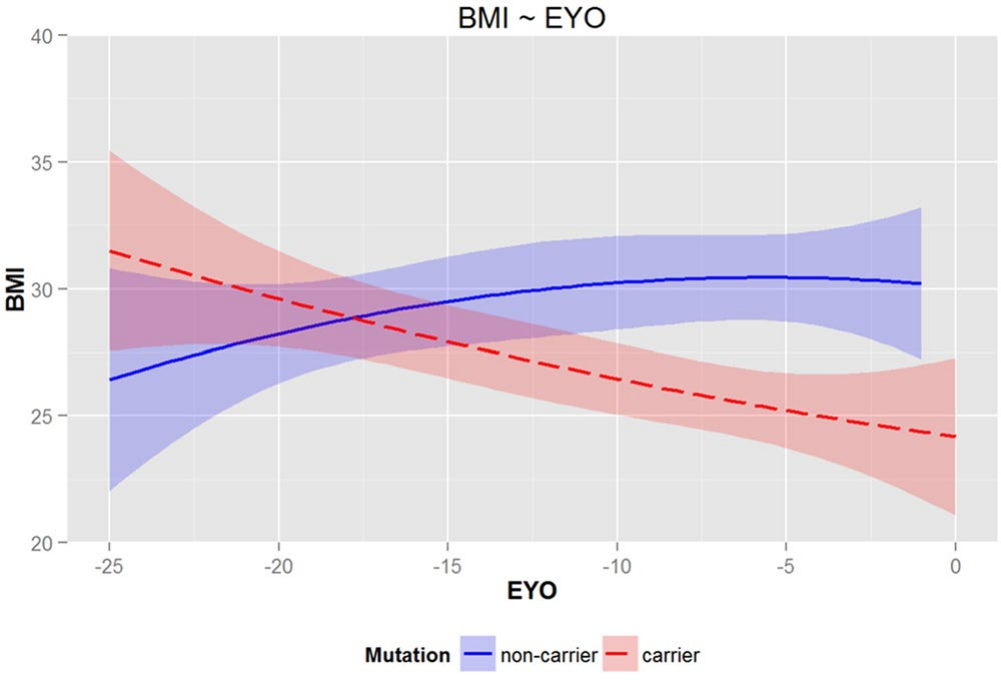
Cross sectional data (LOESS regression analysis and the estimates and their 95% confidence limits were drawn) of body-mass index (BMI) in preclinical (CDR = 0) mutation carriers (MCs) and non-mutation carriers (NCs) in relation to the estimated years to onset (EYO). A significant difference in BMI between MCs and NCs is detected in the area where the estimated 95% confidence intervals for the regression lines don’t overlap (−11.2 years). The intersection points of the lower and upper confidence interval borders were calculated by a numerical approximation. This method was also used to detect the point, at which the two curves begun to diverge (−17.8 years). The use of scatter plots is not permitted with DIAN data as the data points can be very specific to an individual and may allow to identify its mutation status.

In MCs, we found a significant correlation (R = 0.349; P = 0.001) of BMI with EYO (i.e. the lower BMI the closer the years expected to symptom onset) whereas in NC individuals a negative association between BMI and EYO (i.e. BMI slightly increases the closer the years expected to symptom onset) could be detected (R = −0.195; P = 0.037; Table 3). The point at which BMI differs significantly between MCs and NCs was found at 11.2 years (P = 0.012) before expected symptom onset (Fig. 1). Additionally, the two curves begun to diverge at 17.8 years before the expected symptom onset.

**Table 3:** Association between body mass index (BMI) and cognition, and imaging parameters in preclinical (CDR = 0) mutation carriers (MCs) and non-mutation carriers (NCs).

	Outcome	β Coefficient (95% CI)	p value
MC	EYO	−0.263 (−0.552 to −0.026)	0.001
	MMSE	−0.012 (−0.057 to 0.034)	0.895
	Global PIB-uptake	−0.031 (−0.068 to 0.006)	0.032
	LogMem II	1.724 (0.456 to 2.992)	0.017
NC	EYO	0.271 (−0.052 to 0.593)	0.037
	MMSE	0.012 (−0.030 to 0.055)	0.969
	Global PIB-uptake	−0.001 (−0.003 to 0.002)	0.0434
	LogMem II	0.402 (−0.725 to 1.528)	0.397

Note: Results are for linear regression. BMI was the predictor, adjusted for participant age, education and gender. Log Mem II = Logical Memory test, delayed recall; Global PIB-uptake = global cerebral Aβ burden as measured by ^11^C-Pittsburgh Compound-B PET.

No significant correlations were observed in MCs between BMI and MMSE (R = −0.014; P = 0.895) and age (R = −0.175; P = 0.101; Table 3). However, lower BMI was associated with higher amyloid pathology in the brain (R = −0.269; P = 0.032) and lower memory performance (i.e. delayed total recall in the logical memory test (R = 0.253; P = 0.017). As expected, there was a significant correlation in MC participants between EYO and delayed recall of the logical memory test (R = 0.345; P < 0.0001) (i.e. the closer the years expected to symptom onset the more memory impairment).

In NCs no significant correlations between BMI, age (R = 0.211; P = 0.024), global cerebral Aβ burden (R = −0.093; P = 0.434), MMSE (R = −0.004; P = 0.969), and delayed total recall in the logical memory test (R = 0.091; P = 0.397; Table 3) could be observed.

## Discussion

The current study, for the first time, examined the relationship between BMI, clinical and cognitive as well as brain imaging markers of AD in asymptomatic MCs and NCs of families with ADAD. All participants included in the present study showed no relevant signs of cognitive impairment that interfere with activities of daily living as indicated by a CDR score of zero.

The main findings were as follows: (1) Preclinical MCs showed weight loss starting one to two decades before expected symptom onset of ADAD; (2) In preclinical MCs, BMI was significantly correlated with EYO, whereas in NCs a negative association between BMI and EYO could be observed. Thus, while MCs show the lower BMI the closer estimated symptom onset NCs showed evidence for an increase with older age.

Previous studies examining the association between BMI and AD risk have all been performed in elderly people who were cognitively normal or already presented signs of MCI or mild AD^4, 17–19^. In these studies, decreasing body weight has been found to be associated with rapid cognitive decline, AD-like changes in the CSF, and increased risk of developing dementia in elderly individuals^2, 4, 6, 17, 18, 20^. From these studies it has been suggested that BMI alterations may reflect a systemic response to AD-related neuropathology. In addition, some reports suggest that mild weight loss in the elderly may be a downstream effect of normal aging processes associated with reduced metabolic demands, appetite, and diminishing physical stature and height^19, 21^. Thus, it is still unclear to what extent BMI alterations in AD can be attributed to vascular and metabolic comorbidities frequently present in elderly populations and to what extend to “pure” AD.

To address these research questions and to circumvent some of the limitations faced by previous studies, we examined cross-sectional BMI in demographically well-balanced asymptomatic members of families with ADAD. This study allowed us to examine BMI in individuals beginning more than 20 years prior to the EYO and in the absence of clinically relevant impairment in cognitive and functional performance (i.e. CDR = 0) or confounding health effects of aging. We found a significantly lower BMI in MCs compared with NCs in the preclinical stage of ADAD which is consistent with findings of decreased BMI in the preclinical stage of sporadic AD^6, 20, 22, 23^. It should be noted that in the present study, the mean BMI in preclinical MCs and NCs was in the pre-obese range (BMI 25.0–29.9). As expected, NCs showed a significantly higher rate of hypertension which may be due to their higher BMI^24^. However, the divergence in BMI development between MCs and NCs starting one to two decades before symptom onset in the present study is hardly driven by the greater prevalence of obesity (BMI > 30.0) among the NCs alone. It is possibly due to a combination of lower mean BMI in the MC group and increased mean BMI in NCs. However, within-person longitudinal data are warranted to validate this assumption.

The exact causes of lower BMI in the preclinical stage of ADAD are still unknown. Lower BMI in MCs cannot be simply explained as a consequence of dementia syndrome since our study participants were in the preclinical stage of AD with no relevant clinical symptoms of cognitive deterioration. Therefore, low BMI is not expected to be resulting from cognitive impairment. Other possible explanations could be reduced appetite/food intake due to decreased olfactory perception in the early phases of AD making the food less appealing and AD-related brain changes resulting in disturbed food intake and energy homeostasis^22, 25–27^. Recent findings in a transgenic mouse model of AD suggest that energy deficiency due to AD pathology-associated hypermetabolism causes weight alterations^28^. Alternatively, BMI changes might be driven by Aβ accumulation affecting hypothalamic leptin signaling early in the disease process that leads to weight loss and pathologically low leptin state that progressively worsens as the amyloid burden increases^28–30^. In the present study, lower BMI was associated with higher amyloid load in the brain in MC group. This result is in line with previous studies demonstrating an association between BMI and amyloid pathology in the brain and CSF in patients with sporadic AD or in apolipoprotein E (APOE) ε4 alleles carriers as well as in cognitively normal and MCI individuals, suggesting systemic changes in the earliest phases of the disease^20, 31^. The distribution of AD pathology is widespread and accumulates in both cortical and subcortical regions of the brains, which are involved in the maintenance of body composition through effects on appetite or feeding control^20, 22, 25, 32^. Taken together, our findings support the hypothesis that the deposition of AD pathology may contribute to the reduction in body weight prior to the onset of clinical dementia.

Another focus of interest is the association between BMI and cognitive functions. Previous research indicates that both low and high BMI scores have been associated with poorer cognitive functions^33^. In the present study, BMI in preclinical MCs was associated with decreased cognitive performance that was still within normal ranges. The fact that a measurable difference in memory function could be determined between the MC and NC subjects indicates some - if slight - degree of cognitive difference, even years before clinical dementia.

A limitation of this study was that we analyzed cross-sectional baseline data of the DIAN study, with limited predictive power concerning the EYO as opposed to individual longitudinal data. Thus, larger group sizes with longitudinal data are required to accurately ascertain an estimate of the number of years prior to symptom onset when BMI differences emerge between MCs and NCs. However, no sufficient longitudinal data are as yet available to inform on the trajectory of BMI change during preclinical ADAD and as such, our findings should be treated with caution. It should also be noted that results in the early onset group may not be generalizable to those with late onset or sporadic AD, given the potential interaction of mid-life factors, prevalence of co-pathologies, or even substantial different neuropathological mechanisms on weight regulation, as well as the survival effect in the older cohort. Furthermore, although BMI is often used to estimate overweight and obesity, other measures such as waste circumference can provide more accurate measures of body fatness because some individuals having a normal BMI may already have high levels of abdominal obesity, which is considered pro-inflammatory and a risk factor for AD^34, 35^.

In conclusion, the study provides evidence that weight loss starts one to two decades before expected symptom onset of ADAD. Our findings point toward a link between the pathophysiology of ADAD and disturbance of weight control mechanisms. Longitudinal follow-up studies are needed to investigate BMI changes over time.
